# Relation of body mass index to risk of developing inflammatory bowel disease amongst women in the Danish National Birth Cohort

**DOI:** 10.1371/journal.pone.0190600

**Published:** 2018-01-24

**Authors:** Michael Mendall, Maria Christina Harpsøe, Devinder Kumar, Mikael Andersson, Tine Jess

**Affiliations:** 1 Department of Gastroenterology, Croydon University Hospital, Thornton Heath, Surrey, Croydon, United Kingdom; 2 St George’s Medical School, Cranmer Terrace, Tooting, London, United Kingdom; 3 Department of Epidemiology Research, Statens Serum Institut, Copenhagen, Denmark; Dasman Diabetes Institute, KUWAIT

## Abstract

**Background:**

Crohn’s disease (CD) has traditionally been associated with weight loss and low BMI, yet paradoxically obesity has recently been suggested as a risk factor for CD, but not for ulcerative colitis (UC). We therefore hypothesized that the relation between BMI and CD is U shaped.

**Aim:**

To conduct a large population-based prospective cohort study of BMI and later risk of IBD, taking age at IBD diagnosis into account.

**Methods:**

A cohort of 74,512 women from the Danish National Birth Cohort, with BMI measured pre-pregnancy and 18 months after delivery, was followed for 1,022,250 person-years for development of IBD, according to the Danish National Patient Register. Associations were tested by Cox regression.

**Results:**

Overweight subjects (25≤BMI<30 kg/m2) had the lowest risk of CD, whereas obesity (BMI≥30kg/m2) increased the risk of CD at all ages, and low BMI (BMI<18.5kg/m2) associated with CD diagnosed at age 18-<40 years. Hence, using normal weight subjects as the reference, adjusted HRs for risk of developing CD (at age 18-<40 years) were 1.8(95%CI, 0.9–3.7) for underweight, 0.6(0.3–1.2) for overweight, and 1.5(0.8–2.7) for obese individuals (pre-pregnancy BMI). HRs were greater for BMI determined 18 months after delivery. Splines for CD risk according to waist:height ratio confirmed a U-shaped relationship with CD occurring <40 years, and a linear relationship with CD diagnosed at age 40+. There was no relationship between BMI and risk of UC.

**Conclusion:**

For the first time, we demonstrate that both high BMI and low BMI are risk factors for CD. Underweight may be a pre-clinical manifestation of disease being present many years before onset with obesity being a true risk factor. This raises the question as to whether there may be two distinct forms of CD.

## Background

Traditionally Crohn’s disease(CD) has been associated with weight loss and underweight, which are common in newly diagnosed subjects with CD particularly those diagnosed at a young age[1,2]. Paradoxically obesity, as well as at the time diagnosis, has also recently been associated with future risk of Crohn’s disease(CD), but not UC[3,4]. A number of biologically plausible mechanisms exist to explain a causal association between obesity and CD[5].

In an initial case control study, underweight and obese subjects were over-represented at diagnosis. Obese subjects tended to be diagnosed at older ages and the underweight mainly at younger ages. In subjects with UC there was no association[3]. A U-shaped relation between body mass index(BMI) and risk of incident CD was further suggested in an analysis of the relation between BMI and autoimmune diseases from the Danish National Birth Cohort(DNBC)[6]. The analysis was not fully adjusted and this question was not formally addressed.

There is a growing awareness that there may be a prolonged pre-clinical phase to Crohn’s disease[7]. Low BMI rather than being a true risk factor could be a manifestation of this pre-clinical phase, although this has never previously been formally assessed.

The Nurse’s Health Study(NHS)[4] whilst confirming an association between obesity and future risk of CD but not UC, did not demonstrate an association with low BMI, although there was a suggestion of such a relationship in BMIs reported soon before diagnosis. The median age at diagnosis of CD was well over the age of 40 in this study, whereas the median age in the DNBC is lower, the majority of patients being diagnosed under the age of 40.

We aimed to extend the follow-up time of the DNBC cohort and conduct detailed analyses to investigate whether obesity is independently associated with future risk of CD, whether low BMI is independently associated with future development of CD, and whether the relationship between BMI and risk of development of CD is truly U-shaped. Taking advantage of the fact that the DNBC unlike the NHS includes a high proportion of Montreal A2(age at diagnosis 17-<40) patients as well as A3(age at diagnosis 40+), we speculated that a U-shaped relationship would be most clearly manifested in the A2 group, as opposed to the A3 group.

## Methods

### Study population

The study population consisted of women participating in the DNBC recruited by general practitioners in early pregnancy during 1996–2002. The women were interviewed in 16^th^ (interview I) and 32^nd^ (interview II) week of gestation and 6(interview III) and 18 (interview IV) months after delivery (www.dnbc.dk). Originally, 91,767 women were enrolled in the DNBC. Inclusion in the study cohort required a full-term (≥259 days of gestation) live born singleton and that the woman was alive half a year after birth corresponding to start of follow up. BMI had to be within reasonable limits (15≤BMI≤56 kg/m^2^) and information on the potential confounding variables of parity, smoking, alcohol, exercise, and socio-occupational status had to be available at the 6 month post pregnancy interview(missing in n = 1030). Finally, the women were excluded if already diagnosed with IBD at start of follow up (n = 620) resulting in a cohort of 74,512 Danish women. To note and as explained below, BMI at 18 months after delivery was also used as exposure in the same way as pre-pregnancy BMI. This was recorded in interview IV.

### Exposure

In order to calculate pre-pregnancy BMI and BMI 18 months after delivery, questions concerning height (“How tall are you?”) and weight (“What was your weight before the pregnancy?”) from interview I and IV, respectively, were used. Self-reported heights and weights have been shown to be reliable[8]. BMI pre-pregnancy and 18 months post-delivery were chosen so that short-term changes associated with pregnancy which would still be manifest at the 6 month post-pregnancy interview were avoided. The pre-pregnancy BMI gives a measure of body habitus in young adults prior to pregnancy and the 18 months after delivery a better measure of BMI though later adult life given the long-term changes in BMI which can occur following pregnancy. The BMI ranges used were based on the international WHO classification where BMI<18.5 kg/m2 is considered underweight, 18.5-<25 kg/m2 normal weight(reference group), 25-<30 kg/m2 overweight and ≥30 kg/m2 obese[9].

Waist:height ratio was determined in a limited number of women 18 months after birth using self-reported waist measurement[10].

### Outcomes

Linkage to registers were done using a 10-digit unique personal identification number registered in the Danish Civil Registration System[11] given to all Danish citizens at birth. Information on diagnoses of the inflammatory bowel diseases, CD and UC was obtained from the Danish National Patient Register[12] using codes from the International Classification of Diseases, 8^th^ (ICD-8) and 10^th^ (ICD-10) revision. Diagnoses on in- and out patients were included (CD [ICD-8: 563.0 and ICD-10: K50] and UC [ICD-8: 563.19, 569.04 and ICD-10: K51]). ICD-8 codes were used to exclude prevalent IBD before start of follow up. Patients who shifted diagnosis from one IBD to the other counted as having the latest recorded diagnosis but were included as cases from the date of first IBD diagnosis. Only patients with more than one IBD diagnosis or admission in the hospital for more than a week were counted as cases. Patients with IBD diagnoses who did not fulfill this criteria were censored at the date of the diagnosis. This approach has previously been validated(using a pathology database as reference) in the National Registry, with the proportion of confirmed cases being 97% for CD and 90% for UC[13]

### Covariates

Confounding variables chosen a priori were obtained from the DNBC. These included alcohol consumption per week prior to pregnancy (0 units, 1–7 units, ≥8 units), socio-occupational status at pregnancy (based on the woman’s current or most recent occupation within the last six months, or if in school, on type of education; 1. Long education or leaders in large companies; 2. Middle long education or leaders in small companies; 3. Short education, vocational, or under education, 4. Unskilled, other work, or receiving unemployment benefits; 5. On state welfare; variable defined previously [14] parity at pregnancy (0 children, 1 child, ≥2 children), and ever smoking (yes/no; during and/or after pregnancy reported in interview I, II, and/or III versus no smoking reported in any of the assessed interviews; information on smoking status prior to pregnancy is not available in the interviews). Exercise data was collected in interview 1 and relates to exercise in the first part of pregnancy divided into no exercise/week, 1-<120 min/week, 120-<240 min/week, 240-<420 min/week and 420+ min/week. There is no information on exercise prior to pregnancy. Further, risk factors (yes/no) used in the analyses and obtained from the Danish Prescription Register and National Patient Register were: appendectomy (KJEA, 43000, 43001 and 45100), gestational, type 1 and 2 diabetes mellitus (ICD-8: 249.00–250.99 and 634.74, ICD-10: E10-14, D024.4, DO240, DO241, DO243 and DO245), oral contraceptives prior to pregnancy (ATC-code: G03A), use of proton-pump inhibitors (PPIs: Omeprazole, Lansoprazole, Esomeprazole, Pantoprazole, Rabeprazole; ATC-code: A02BC) and being vegetarian (Interview II). Regarding appendectomy, diabetes and PPIs, these had to be registered before start of follow up.

### Statistical analyses

Hazard ratios (HRs) with 95% confidence intervals (CIs) were calculated using Cox regression. Women were followed (using pre-pregnancy BMI from interview I) or 18 months after delivery (using BMI from interview IV) until CD or UC development, emigration, death, or end of follow-up, 31st of December, 2014 with the woman’s age as underlying timescale. Follow-up started from the 6 months post-pregnancy interview. The women only contributed with time while registered as living in Denmark (i.e. women moving away from Denmark for a time period but later moved back contributed with time in both periods living in Denmark). Similar analyses were performed for the two different BMI exposures. Alcohol, socio-occupational status and parity were accounted for by including them as strata variables in the Cox regression whereas smoking and risk factors were adjustment variables fixed at baseline. Exercise was excluded as a confounder in the final results since it showed an effect of less than 3% on estimates, did not seem related to either risk of disease or to BMI unlike the other covariates, and to increase power.

Cubic splines restricted to be linear in the tails were constructed to visualize a potential linear or U-shaped relationship between risk of IBD, BMI and in a limited number of subjects waist:height ratio. The splines were estimated for both the total cohort and for age-groups (with 40 years of age as cutting point). No violation of the proportional hazards assumption was found evaluated by the empirical score process[15]. All statistical tests were evaluated by Wald test statistics using SAS software version 9.4 (SAS Institute Inc., Cary, NC, USA).

We additionally divided the women into either smokers or no indication of smoking to evaluate whether smoking had an effect on estimates especially in the underweight category.

## Results

The main cohort of 74,512 women (the sub-cohort of women with BMI 18 months measurements consisted of 48,262 women) were followed for a median of 13.9 years (lower-upper quartile: 12.7–15.0). In Table 1, characteristics of the cohort are shown. In the pre-pregnancy group, 8.1% were obese and 4.4% were underweight, and in the 18 months postpartum group, 7.7% were obese and 4.3% were underweight. Compared with the normal weight group of women, more obese women seemed to have diabetes, PPI use, appendectomy, shorter education whereas obese women smoked less and drank less alcohol. There were no clear differences in the amount of exercise taken between groups. Table 2 shows the time from inclusion in the study to the development of CD and UC with no evidence of clustering of cases near the beginning of follow-up.

**Table 1:** Cohort characteristics by BMI group (n = 74,512 pre-pregnancy / 48,262 18 months after delivery).

***Body mass index kg/m***^***2***^	*<18*.*5*	*18*.*5-<25*	*25-<30*	*≥30*
**Person years at risk**	*45*,*305*	*694*,*405*	*196*,*256*	*81*,*737*
**Distribution of pre-pregnancy BMI *(n [%])* **	3302 (4,4)	50,776 (68,1)	14,403 (19,3)	6031 (8,1)
**Distribution of BMI 18 months postpartum (n [%]) **	2095 (4,3)	32,473(67,3)	9933 (20,6)	3729 (7,7)
**Age at entry** *(median years [lower-upper quartiles])*	30,0 (27.1:33.3)	30.8 (28.1:33.9)	30.5 (27.7:33.6)	30.3 (27.4:33.3)
**Alcohol consumption per week prior to pregnancy** *(n [%])*				
0 units	555 (16.8)	5428 (10.7)	2101 (14.6)	1274 (21.1)
1–7 units	2466(74.7)	39604(78.0)	11122(77.2)	4421 (73.3)
8+ units	281 (8.5)	5744 (11.3)	1180 (8.2)	336 (5.6)
**Socio-occupational status in pregnancy** *(n [%])*				
Long edu/leaders in large companies	680 (20.6)	11990 (23.6)	2303 (16.0)	620 (10.3)
Middle long edu/leaders in small companies	879 (26.6)	16987(33.5)	4344 (30.2)	1536 (25.5)
Short edu/vocational/under edu	1314(39.8)	17787(35.0)	6187 (43.0)	2869 (47.6)
Unskilled/other work/unemployment benefits	305 (9.2)	3081 (6.1)	1250 (8.7)	791 (13.1)
On state welfare	124 (3.8)	931 (1.8)	319 (2.2)	215 (3.6)
**Parity prior to pregnancy** *(n [%])*				
0 children	1618 (49.0)	25613 (50.4)	6691 (46.5)	2784 (46.2)
1 child	1219 (36.9)	17360 (34.2)	5269 (36.6)	2213 (36.7)
2+ children	465 (14.1)	7803 (15.4)	2443 (17.0)	1034 (17.1)
**Smoking ever, during, or after pregnancy** *(n [%])*				
Smoking reported in interview I, II, or III	1252(37.9)	13729(27.0)	3919 (27.2)	1612 (26.7)
No smoking reported in interview I, II, or III	2050(62.1)	37047(73.0)	10484(72.8)	4419 (73.3)
**Appendectomy** *(n [%])*				
Yes	246 (7.5)	3693 (7.3)	1146 (8.0)	525 (8.7)
No	3056(92.5)	47083(92.7)	13257(92.0)	5506 (91.3)
**Diabetes mellitus** *(n [%])*				
Yes	4 (0.1)	106 (0.2)	65 (0.5)	53 (0.9)
No	3298(99.9)	50670(99.8)	14338(99.5)	5978 (99.1)
**Oral contraceptive use before pregnancy** *(n [%])*				
Yes	2427 (73.5)	37496 (73.8)	10847 (75.3)	4503 (74.7)
No	875 (26.5)	13280 (26.2)	3556 (24.7)	1528 (25.3)
**Proton pump inhibitor use***(n [%])*				
Yes	161 (4.9)	1636 (3.2)	556 (3.9)	320 (5.3)
No	3141(95.1)	49140(96.8)	13847(96.1)	5711 (94.7)
**Vegetarian** *(n [%])*				
Yes	71 (2.3)	676 (1.4)	91 (0.7)	32 (0.6)
No	2995 (97.7)	47139 (98.6)	13515 (99.3)	5638 (99.4)
**Exercise(n(%)** No exercise	2233(67.6)	30923(60.9)	9346(64.9)	4107(68.1)
1-<120mins/week	555(16.8)	10714(21.1)	2869(19.9)	1139(18.9)
120-<240min/week	329(10.0)	5998(11.8)	1523(10.6)	552(9.2)
240-<420min/week	129(3.9)	2332(4.6)	479(3.3)	156(2.6)
420+mins/week	56(1.7)	809(1.6)	186(1.3)	77(1.3)

**Table 2:** Time from inclusion in the study to the development of CD and UC with no evidence of clustering of cases near the beginning of follow-up.

Years_since_entry	Outcome_type		
Frequency	CD(column percent)	UC(column percent)	Total
**0**	11(8.03)	39(8.71)	50
**1**	6(4.38)	28(6.25)	34
**2**	12(8.76)	52(11.61)	64
**3**	13(9.49	32(7.14)	45
**4**	8(5.84)	47(10.49)	55
**5**	11(8.03)	23(5.13)	34
**6**	10(7.30)	28(6.25)	38
**7**	12(8.76)	29(6.47)	41
**8**	7(5.11)	33(7.37)	40
**9**	6(4.38)	24(5.36)	30
**10**	12(8.76)	26(5.80)	38
**11**	10(7.30)	29(6.47)	39
**12**	6(4.38)	27(6.03)	33
**13**	8(5.84)	21(4.69)	29
**14+**	5(3.65)	10(2.23)	15
**Total**	137	448	585

### Risk of CD and UC

During 1,022,250 years of follow-up, 137 women (0.2%) developed CD and 448 (0.6%) developed UC.

Table 3 shows the relationship between BMI (pre-pregnancy, respectively, 18 months after delivery) and risk of developing CD and UC, for the whole cohort and divided into those developing IBD age18-<40 and 40+. With CD, for BMI pre-pregnancy, in the total population the age adjusted HR was 2.1(95%CI, 1.1–3.9) for low BMI(<18.5). 0.9(0.6–1.5) for overweight(BMI 25+-<30) and 1.7(1.0–2.8) for the obese (BMI>30). For BMI 18 months after delivery, the age adjusted HRs were 2.1(0.9–4.6) for low BMI, 0.6(0.3–1.2) for overweight and 2.0(1.1–3.8) for the obese. At both time-points the overweight had the lowest risk. As with BMI pre-pregnancy, there was very little attenuation of the effect size for the obese after adjustment, but there was modest attenuation of the effect size associated with low BMI, the HRs for low BMI being 1.4(0.7–2.9), 0.8(0.5–1.4) for overweight and for the obese 1.5(0.9–2.6) pre-pregnancy and 1.6(0.7–3.7). 0.6(0.3–1.1) and 1.9(1.0–3.7) for BMI 18 months after delivery. No clear association with BMI was discernible for UC.

**Table 3:** Hazard ratios (HRs with confidence intervals) of development of CD or UC according to body mass index (BMI; kg/m^2^) overall and divided into age groups before and after 40 years of age.

	No of persons	PYRFU#	No of cases in total	No of cases < 40 years	No of cases ≥ 40 years	Unadjusted HR	Adjusted HR @	Unadjusted HR < 40 years	Adjusted HR < 40 years @	Unadjusted HR ≥ 40 years	Adjusted HR ≥ 40 years @
***Crohn’s disease***											
**Pre-pregnancy BMI **											
BMI<18.5	3302	44982.3	12	11	1	2.1 (1.1–3.9)*	1.4 (0.7–2.9)	2.5 (1.3–4.7)*	1.8 (0.9–3.7)*	0.8 (0.1–5.8)	0.0 (0.0-)
18.5≤BMI<25	50776	690743.2	85	63	22	1 (Ref.)	1 (Ref.)	1 (Ref.)	1 (Ref.)	1 (Ref.)	1 (Ref.)
25≤BMI<30	14403	195682.4	23	14	9	0.9 (0.6–1.5)	0.8 (0.5–1.4)	0.8 (0.4–1.4)	0.6 (0.3–1.2)	1.5 (0.7–3.3)	1.5 (0.7–3.2)
BMI≥30	6031	81580.4	17	12	5	1.7 (1.0–2.8)	1.5 (0.9–2.6)	1.5 (0.8–2.8)	1.5 (0.8–2.7)*	2.1 (0.8–5.6)	1.6 (0.6–4.9)
**BMI 18 after delivery**											
BMI<18.5	2095	26645.3	7	7	0	2.1 (0.9–4.6)*	1.6 (0.7–3.7)*	2.8 (1.2–6.3)*	2.1 (0.9–5.0)*	0.0 (0.0-.)	0.0 (0.0-.)
18.5≤BMI<25	32473	410895.7	51	36	15	1 (Ref.)	1 (Ref.)	1 (Ref.)	1 (Ref.)	1 (Ref.)	1 (Ref.)
25≤BMI<30	9933	125630.2	9	2	7	0.6 (0.3–1.2)	0.6 (0.3–1.1)	0.2 (0.0–0.7)	0.2 (0.0–0.7)	1.6 (0.6–3.9)	1.5 (0.6–3.7)
BMI≥30	3729	46880.7	12	9	3	2.0 (1.1–3.8)*	1.9 (1.0–3.7)*	2.0 (1.0–4.2)*	1.9 (0.9–4.0)*	2.0 (0.6–6.8)	1.9 (0.5–6.9)
											
***Ulcerative colitis***											
**Pre-pregnancy BMI **											
BMI<18.5	3302	44982.3	25	18	7	1.2 (0.8–1.9)	1.1 (0.7–1.7)	1.2 (0.8–2.0)	1.1 (0.7–1.9)	1.3 (0.6–2.7)	1.0 (0.4–2.3)
18.5≤BMI<25	50776	690743.2	308	212	96	1 (Ref.)	1 (Ref.)	1 (Ref.)	1 (Ref.)	1 (Ref.)	1 (Ref.)
25≤BMI<30	14403	195682.4	85	59	26	1.0 (0.8–1.2)	0.9 (0.7–1.2)	1.0 (0.7–1.3)	1.0 (0.7–1.3)	1.0 (0.7–1.6)	0.9 (0.5–1.4)
BMI≥30	6031	81580.4	30	20	10	0.8 (0.6–1.2)	0.7 (0.5–1.1)	0.8 (0.5–1.2)	0.7 (0.5–1.2)	1.0 (0.5–1.9)	0.7 (0.4–1.5)
**BMI 18 months after delivery**											
BMI<18.5	2095	26645.3	13	8	5	1.0 (0.6–1.8)	1.0 (0.6–1.7)	0.9 (0.4–1.8)	0.8 (0.4–1.7)	1.4 (0.5–3.4)	1.4 (0.5–3.4)
18.5≤BMI<25	32473	410895.7	194	130	64	1 (Ref.)	1 (Ref.)	1 (Ref.)	1 (Ref.)	1 (Ref.)	1 (Ref.)
25≤BMI<30	9933	125630.2	46	26	20	0.8 (0.6–1.1)	0.7 (0.5–1.0)	0.6 (0.4–1.0)	0.6 (0.4–1.0)	1.1 (0.6–1.8)	1.0 (0.6–1.7)
BMI≥30	3729	46880.7	20	13	7	0.9 (0.6–1.4)	0.8 (0.5–1.3)	0.8 (0.5–1.4)	0.7 (0.4–1.3)	1.1 (0.5–2.4)	0.9 (0.4–2.2)

# Patient years of follow-up.

@ Adjusted for smoking, appendectomy, socio-economic status, parity, diabetes, oral contraceptive pill use, proton-pump inhibitor, vegetarian diet, and alcohol consumption. Exercise was excluded as it had no association with risk of disease or BMI and merely reduced power.

* p<0.05 v overweight category.

**Fig 1:**
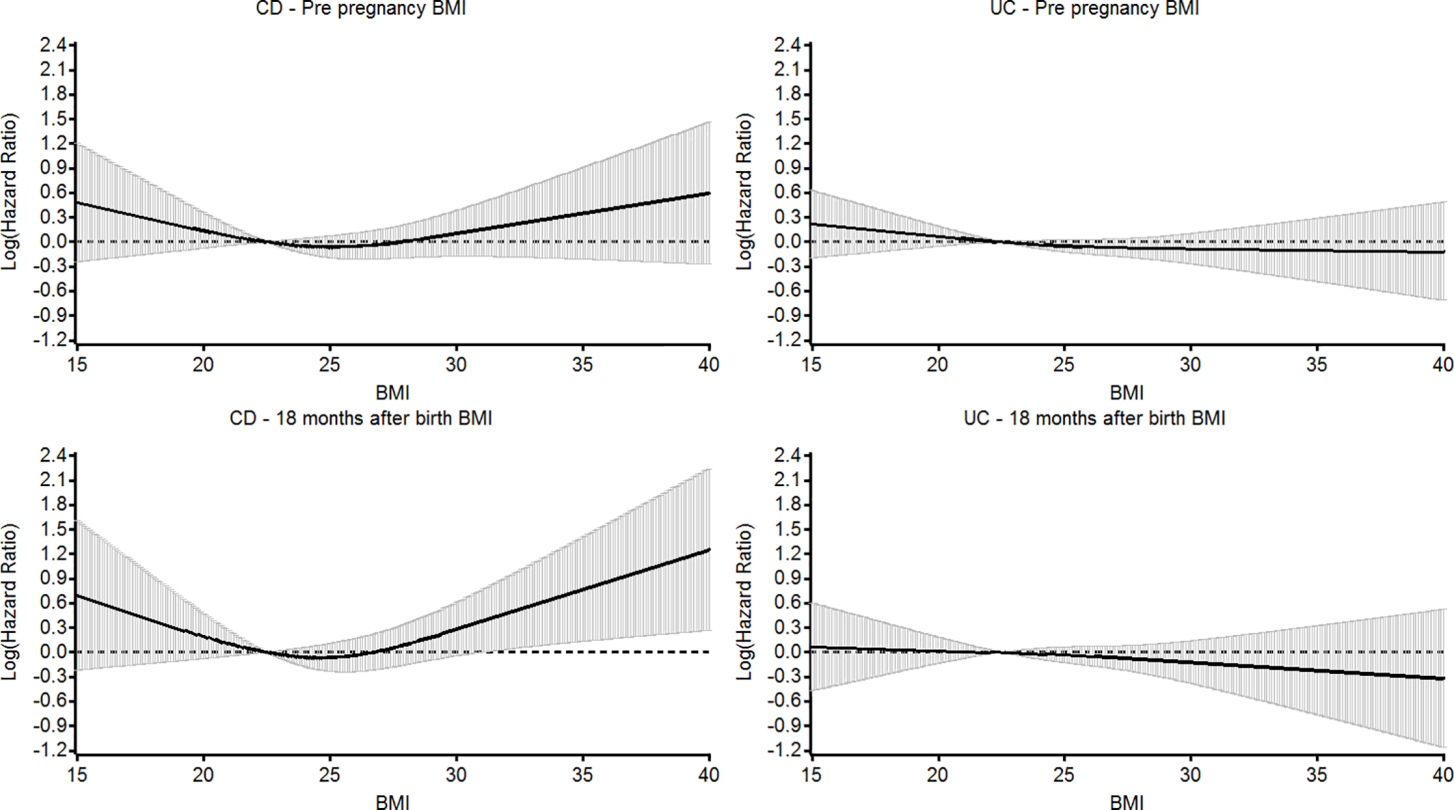
Cubic splines of BMI versus hazard ratio for the development of Crohn’s disease and ulcerative colitis in both age groups combined for pre-pregnancy BMI and BMI 18 months after delivery. For pre-pregnancy BMI for the CD spline p = 0.2777 for UC 0.4146. For 18 months after delivery for CD p = 0.0354 and for UC p = 0.6359. 95% Cis are displayed.

This is reflected in the splines which are presented in Fig 1. For CD, the 18 month after delivery spline displayed a statistically significant U-shape, the U-shape also being present for the pre-pregnancy BMIs but falling short of statistical significance. For UC the splines were not significant and clearly different to those observed for CD.

In Table 3 when subjects diagnosed age18-<40 and 40+ were analyzed separately, the U-shaped relation was more marked in the under 40s and was not present in the over 40s at both BMI time points, mainly because only one low BMI subject at baseline developed CD. Before adjustment, the HRs in the 18-<40s using normal BMI as reference were 2.5(1.2–6.3) for low BMI, 0.8(0.4–1.4) for overweight BMI and 1.5(0.8–2.8) for the obese. There was no attenuation of the HR for obesity but some attenuation of the association with low BMI. At 18 months after delivery the same pattern was observed only more marked, the respective HRs in the 18-<40s were 2.8(1.2–6.3), 0.2(0.0–0.7) and 2.0(1.0–4.2). Again, there was little attenuation of the HR for obesity after adjustment, but modest attenuation of the HR for low BMI. In the 40+ at diagnosis only one subject had low BMI at baseline making estimates unreliable, but there was progressive rise in risk with increasing BMI at both time points from 0.8(0.1–5.8) for low BMI to 2.1(0.8–5.6) for the obese unadjusted pre-pregnancy, with some attenuation after adjustment. A similar pattern was observed for the 18 months after delivery BMI although there were no subjects in the low BMI category.

Compared to the overweight lowest risk group, all differences with low BMI and obesity were significant with the exception of obesity and low BMI pre-pregnancy and both low and high BMI in the 40+ age group.

**Fig 2:**
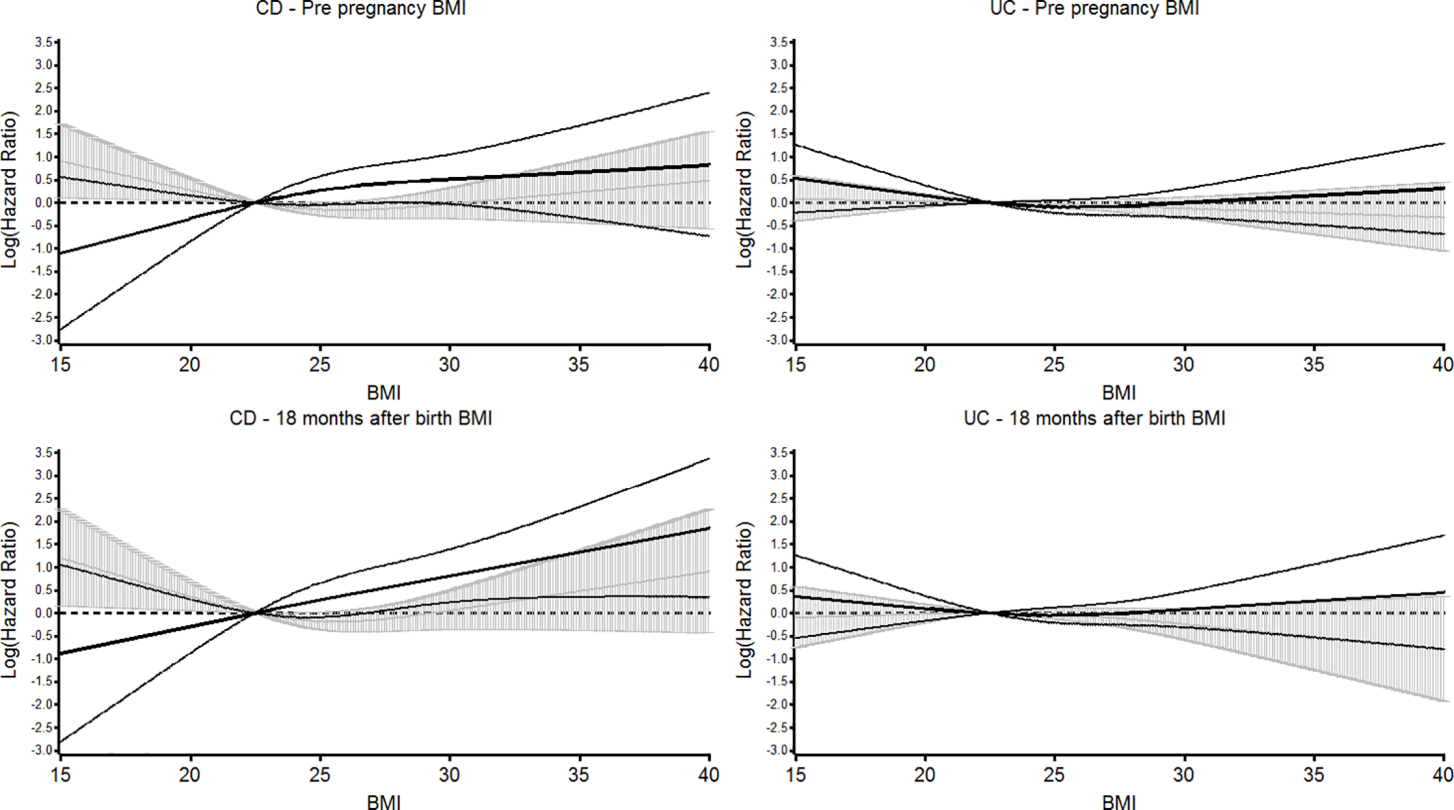
Cubic spines of BMI versus hazard ratio for the development of Crohn’s disease and ulcerative colitis separately in the under and over 40s at time of diagnosis for pre-pregnancy BMI and BMI 18 months after birth with 95% CIs. The probability that the splines for the under and over 40 years of age are different pre-pregnancy = 0.0575, and for the 18 months post delivery p = 0.0618. F or UC the respective probabilities were p = 0.4989 and p = 0.3395. The spline age 18-<40 and 95% Cis are shaded and the spline for age 40+ and 95% Cis are unshaded.

Fig 2 shows splines in the under and over 40s at the time of diagnosis. In the under 40s the splines for CD HR were U-shaped in both the 18 month after delivery and pre-pregnancy, but linear in the over 40s. P values of 0.0575 for the pre-pregnancy BMI and 0.0618 for the 18 months after delivery were obtained on testing whether the splines differed between the two age groups.

There was no significant difference in time to diagnosis by BMI category (Table 4), the median time to development of CD being 7 years for low BMI falling to 5.5 years for the obese. For UC, the respective figures were 7.2 yrs and 5.4 yrs. Median time to development of CD was 5.5 in the under 40’s and 10.4 in the over 40’s, and in UC 4.4yrs and 10.6yrs respectively.

**Table 4:** Time in years from inclusion to development of CD or UC (based on 6 months postpartum).

	Number of cases unadj.	Number of cases adj.	Median years (lower-higher quartile)
***Crohn’s disease***			
**Pre-pregnancy BMI **			
BMI<18.5	12	9	7.0 (2.8–10.0)
18.5≤BMI<25	85	81	6.9 (3.7–10.3)
25≤BMI<30	23	21	6.9 (2.7–11.3)
BMI≥30	17	16	5.1 (3.3–7.6)
Age < 40 years	100	93	5.5 (2.7–8.1)
Age ≥ 40 years	37	34	10.4 (8.2–12.8)
***Ulcerative colitis***			
**Pre-pregnancy BMI **			
BMI<18.5	25	23	7.2 (2.1–10.1)
18.5≤BMI<25	308	293	6.5 (3.1–10.2)
25≤BMI<30	85	80	4.9 (2.5–8.9)
BMI≥30	30	27	5.4 (2.8–10.3)
Age < 40 years	309	294	4.4 (2.3–7.4)
Age ≥ 40 years	139	129	10.6 (8.0–12.7)

### Smoking, BMI and risk of IBD

We determined whether a history of smoking may explain the association between low BMI and risk of CD by performing an analyses restricted to non-smokers and smokers. For pre-pregnancy BMI, the HRs for CD in non-smokers were 2.28 (0.97–5.33), 0.90 (0.48–1.70) and 1.96 (1.02–3.79) for underweight, overweight and obese relative to normal weight subjects, as opposed to 1.65 (0.70–3.91), 1.00 (0.51–1.95), 1.29 (0.55–3.05) in smokers. Likewise, for BMI 18 months after delivery the respective HRs for CD were 1.71 (0.52–5.60), 0.62 (0.26–1.48) and 1.63 (0.68–3.89) in non-smokers and 2.16 (0.73–6.35), 0.49 (0.15–1.66), 2.68 (1.07–6.71) in smokers. Tests for interaction between BMI and smoking for CD were non-significant, p = 0.91 and p = 0.09 for UC for pre-pregnancy BMI. There was no evidence of interaction with oral contraceptive use either p = 0.95 for CD and 0.21 for UC.

### Waist: Height ratio and risk of IBD

**Fig 3:**
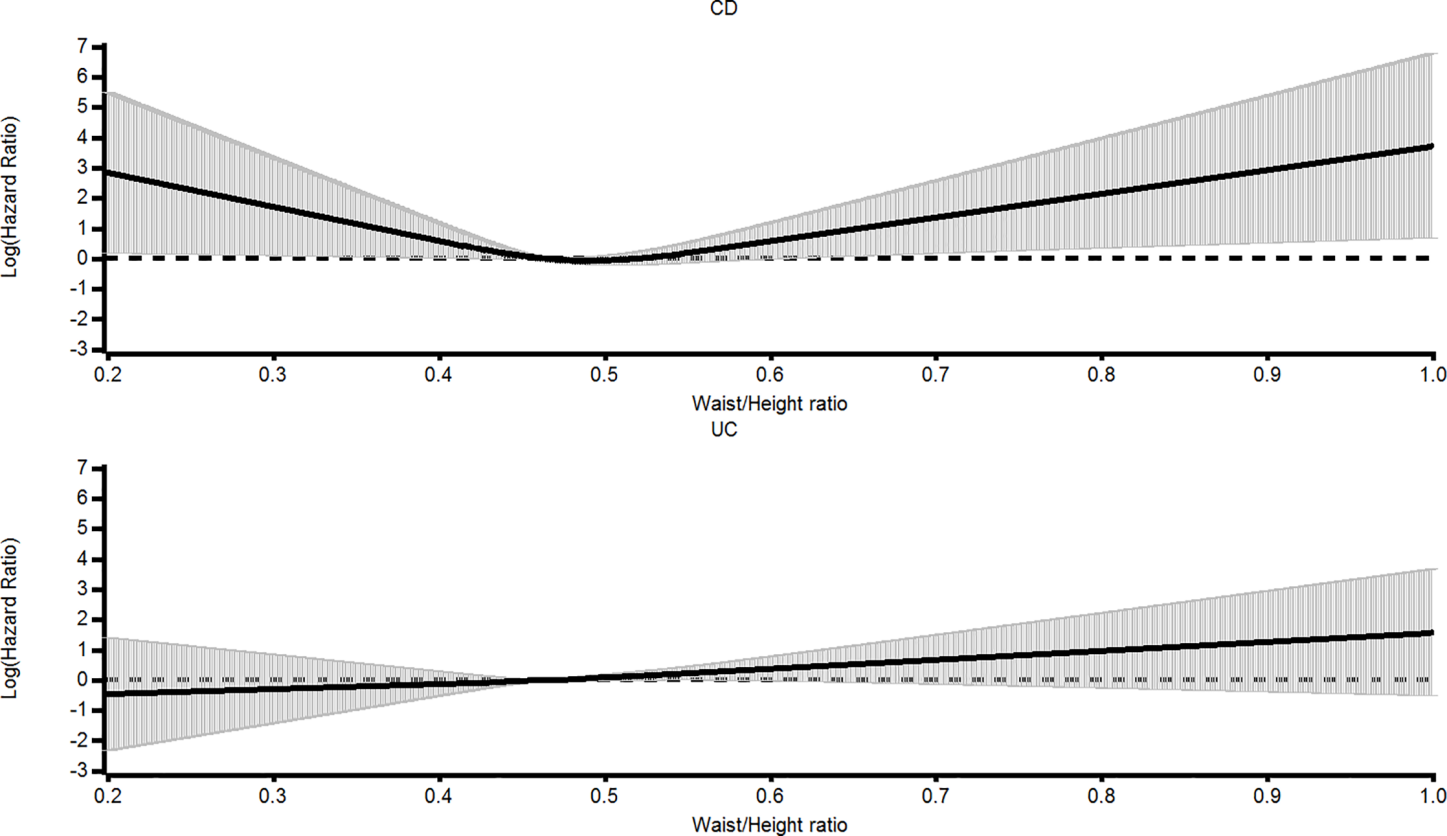
Splines for waist: Height ratio 18 months after delivery and hazard ratios for the development of CD and UC with 95% CIs. For CD spline p = 0.0256 and for UC p = 0152. Test for trend for CD p = 0.9032 and for UC 0.059.

Waist measurements were obtained in 21,014 subjects at 18 months after delivery covering 32 incident cases. For CD, unadjusted HRs for successive quartiles of waist:height ratio were 1.54 (95% CI, 0.60–3.97) for the first quartile, the second quartile was the reference group, 0.57 (0.17–1.93) and 1.42 (0.54–3.73) for the fourth quartile. Adjusted the respective values (there were 2 fewer cases) were 1.41 (0.53–3.75), 1, 0.53 (0.16–1.83) and 1.10 (0.40–3.00). The corresponding spline shown in Fig 3 demonstrates a significant U-shaped impact of waist:height ratio on risk of CD (p = 0.03). For UC, the unadjusted respective HRs were 0.70(0.43–1.14), 0.71(0.44–1.16) and 1.12(0.73–1.73), the splines not being significant although there was a borderline inversely negative trend.

## Discussion

This large population-based cohort study suggests an association between BMI and risk of developing CD, but not UC. Risk of CD was lowest in the overweight suggesting a U-shaped relationship between both pre-pregnancy and 18 after delivery BMI and risk of CD, although findings were not statistically significant after full adjustment relative to normal weight. When the analysis was restricted to patients aged 18-<40 years at diagnosis, the magnitude of the U-shaped pattern was accentuated, mainly because of an increase in the HR associated with low BMI, again with some attenuation after adjustment but remaining significantly greater than the HR for overweight BMI at both time points. For the over 40s at diagnosis the scarcity of subjects with a low BMI prior to diagnosis is notable, there being an apparent non-significant rise in HR with increasing BMI.

The spline of a subset of women with waist:height ratios confirmed a statistically significant U-shaped relationship in line with that observed for BMI. Splines for the 18 month after delivery BMI confirmed a U shaped relationship, whilst the spline for pre-pregnancy although supportive, was not statistically significant. Splines comparing the association of BMI with risk of CD in the 18-<40s and over 40s confirmed with borderline statistical significance that the risk of developing CD according to BMI is different in the two age groups with a U-shaped relation in the former and a linear association in the latter.

A careful comparison with Nurses Health Study (NHS) is warranted as there are similarities, but also some illuminating differences. Our findings mirror those from the NHS[4] regarding the association of obesity with risk of CD with similar effect sizes. Unlike in the present study, low BMI only began to emerge in the NHS as a risk factor in the 2 years prior to diagnosis, but did not reach conventional levels of significance. The median time to the development of CD from baseline was longer in that study-10 years as opposed to just 6 years in the DNBC’s 18-<40s, so that one explanation for the difference may be the closer proximity to developing CD in the current study. However low BMI was a risk factor for CD a median of nearly 9 years prior to diagnosis in the DNBC (the start of follow-up was 6 months post-partum), making this unlikely to be the whole explanation. Another explanation maybe the younger age at entry in the DNBC (median age 30.7, quartiles 27.9,33.8) particularly as the risk associated with low BMI was restricted in the DNBC to the 18-<40’s.

The finding of a lack of association between low BMI with risk of CD solely in the over 40s could be spurious. It may be a chance result of the smaller number of cases (only ¼ of cases were aged 40+). Alternatively, it could be that the longer time to development of IBD in those subjects who developed it over the age of 40 meant that low BMI was not evident at baseline, with a reduced BMI emerging closer to the manifestation of the disease. The time to development of CD was longest in the underweight category and shortest in the obese category argues against this as an explanation. Our previously reported studies also support this explanation: low BMI at diagnosis was mainly apparent in younger subjects[1], and reduction in BMI prior to diagnosis was less in the Montreal A3 group[4].

### Strengths and limitations of the study

The strength of this study is that it is a prospective large population based cohort study with index cases straddling the conventional Montreal classification between A2 and A3, although a sizeable proportion of patients do develop Crohn’s disease at an even earlier age. The cases were well characterized and required at least two clinical contacts to be included as a case. It is possible that some of the cases were misclassified, but this would only have weakened our findings. Another strength is the broad range of risk factors for IBD which were controlled for. Furthermore, the subjects represent the full social strata of Danish society, unlike the NHS.

An important weakness is the relatively small number of cases yielding p values in some instances only just or short of statistical significance particularly after adjustment. This is in the nature of prospective cohort studies for relatively uncommon diseases such as IBD. However, associations were consistent when looked at in a number of different ways. Furthermore the attenuation of effect sizes after adjusting for confounders was modest in the underweight and virtually non-existent for the obese. That our findings for BMI were confirmed on analyzing waist:height ratio adds confidence that the findings have not arisen by chance.

Another weakness of this study is that it is restricted to women who became pregnant. Nevertheless, this represents a sizeable minority of the population whose study should inform us about CD in general. Underweight and obesity can be associated with sub-fertility, and hence the extremes of BMI may be under-represented in this cohort, but if anything this potential source of bias would be expected to diminish the effect sizes observed.

Whilst we were able to control for all the main risk factors for CD we were unable to control in detail for diet apart from vegetarianism. However in the NHS controlling for dietary components did not have any effect on risk estimates. Also some of the risk factors controlled for relate to pre-pregnancy or pregnancy exposure eg oral contraceptive use and exercise, with no information regarding use post pregnancy, whereas others such as smoking status were collected both during and after pregnancy. Again, in the NHS controlling for these risk factors had no effect.

It would have been informative to have the full Montreal classification for these subjects, but this was not available. It would be of particular interest to determine whether the site of the GI tract affected differed with increasing BMI and increasing age at diagnosis. Colonic location for instance becomes more common with increasing age[16] and it may be that this is the location particularly associated with obesity. Information on mode of delivery was also not available.

BMI is a relatively poor measure of the visceral adiposity which is believed to be important in the metabolic and systemic effects of obesity. More specifically in the context of CD, visceral fat area which does not necessarily correlate with BMI, is the best marker of post-operative recurrence[17]. We only had very limited numbers of subjects with waist:height ratio which is a better measure of visceral adiposity[18]. This confirmed our findings and reassuringly there was less evidence of effect size reduction for those with low waist:height ratios then with low BMI after adjustment for confounding factors.

### The interpretation and significance of our findings

A number of mechanisms have now been recognized whereby obesity could contribute to risk of CD. These include alterations in gut permeability[19], elevated levels of bowel inflammation[20], the direct pro-inflammatory effects of free fatty acids on cytokine production[21], epigenetic alterations which are observed in obesity and IBD and which can be induced by fatty acids[22] as well as alterations in gut microbiome[23]. A recently published study sheds light on the latter. A loss of function single nucleotide polymorphism in an intestinal border zinc transporter protein was found to be associated with risk of CD, obesity and with alterations in gut microbiome common to both CD and obesity[24]. The transporter polymorphism was also associated with the metabolic syndrome and hypertension.

Whilst the current study and the NHS do not support confounding of the association of obesity by standard risk factors, the most likely being lack of fibre intake and exercise, some other feature of dietary intake such as fat consumption is a possibility. We cannot discount this.

It is less likely that low BMI is causally related to risk of CD, but rather a sub-clinical early manifestation of the disease longer than the clinical prodrome, which is usually less than 2 years[25]. Although there was some attenuation of the association after controlling, no single factor seemed to play a dominant role in reducing the magnitude of the association. If a causal role for low BMI is not being claimed then effect size reduction by other causally related factors is of lesser importance. A long sub-clinical prodrome is supported by recent observations that serum C-reactive protein and IL-6 are associated with future of risk of disease up to 14 years in the future and the finding that serological markers for CD appear many years before clinical presentation[26]. Alternatively, it may be that an environmental or genetic risk factor associated with both low BMI and risk of CD may be the explanation, the most likely one being smoking. However, we found little evidence of effect reduction in non-smokers and our analysis was adjusted for smoking. Pure ileal disease has the strongest association with CARD mutations which themselves are associated with more severe disease and younger age at onset[27]. It would be of interest to determine whether asymptomatic carriers of these single nucleotide polymorphisms are underweight.

Many factors related to CD pathogenesis have been proposed as playing a role in this weight loss, including the anorectic and catabolic effects of pro-inflammatory cytokines[28], alteration in production of the adipokines leptin and adiponectin[29], dietary restriction to alleviate symptoms[30], and micro-nutrient deficiencies[31]. Why these issues are more prominent in some forms of CD and younger rather older onset is unclear. Younger subjects have a heavier genetic burden as alluded to above, for example with CARD mutations which are associated with more severe disease and weight loss. Another explanation may be different sites of the GI tract affected, colonic involvement with less associated inflammatory response being more common in older subjects[16]. The reason that weight loss may not be an issue for UC could relate to a shorter prodrome or a less pronounced systemic inflammatory response related to its non-transmural nature and lack of involvement of visceral adipose tissue[32].

### Conclusions

This study provides confirmation of the association between obesity and risk of CD and for the first time show that low BMI can precede the development of CD by many years. This finding requires replication in other cohorts and in men. The possibility that there may be two forms of CD—a classical skinny type and an obese phenotype is raised, requiring further investigation into differing pathogenesis and treatment. If weight loss occurs many years before diagnosis early markers of disease are required possibly allowing intervention before clinical presentation. This would offer the hope of preventing the irreversible intestinal damage frequently present when first diagnosed.
